# Nuclear PKM2 contributes to gefitinib resistance via upregulation of STAT3 activation in colorectal cancer

**DOI:** 10.1038/srep16082

**Published:** 2015-11-06

**Authors:** Qiong Li, Daoxiang Zhang, Xiaoying Chen, Lei He, Tianming Li, Xiaoping Xu, Min Li

**Affiliations:** 1 Department of Laboratory Medicine, Renji Hospital, School of Medicine, Shanghai Jiaotong University, Shanghai, 200127, China; 2 Division of Oncology, School of Medicine, Washington University in St. Louis, MO, 63110, USA

## Abstract

Gefitinib (Iressa, ZD-1839), a small molecule tyrosine kinase inhibitor (TKI) of the epidermal growth factor receptor (EGFR) pathway, is currently under investigation in clinical trials for the treatment of colorectal cancer (CRC). However, as known, some patients develop resistance to TKIs, and the mechanisms mediating intrinsic resistance to EGFR-TKIs in CRC have not been fully characterized. Resistance to EGFR inhibitors reportedly involves activation of signal transducer and activator of transcription 3 (STAT3) in glioma and lung cancer. Here, we demonstrated that the nuclear pyruvate kinase isoform M2 (PKM2) levels were positively correlated with gefitinib resistance in CRC cells. The overexpression of nuclear PKM2 in HT29 cells decreased the effect of gefitinib therapy, whereas PKM2 knockdown increased gefitinib efficacy. Furthermore, the activation of STAT3 by nuclear PKM2 was associated with gefitinib resistance. Inhibition of STAT3 by Stattic, a STAT3-specific inhibitor, or STAT3-specific siRNA sensitized resistant cells to gefitinib. These results suggest that nuclear PKM2 modulates the sensitivity of CRC cells to gefitinib and indicate that small molecule pharmacological disruption of nuclear PKM2 association with STAT3 is a potential avenue for overcoming EGFR-TKI resistance in CRC patients.

Colorectal cancer (CRC) is one of the most prevalent malignancies in the world. More than 1.2 million new colorectal cancer cases and 600,000 deaths due to CRC are reported yearly^1^. In the past several decades, the treatment for CRC has evolved to target-specific vehicles and combination cytotoxic therapy rather than single-agent chemotherapy. Gefitinib (Iressa, ZD-1839) is a small molecule tyrosine kinase inhibitor (TKI) targeting the epidermal growth factor receptor (EGFR) signal transduction pathway that is involved in the survival and proliferation of cancer cells. In clinical treatment settings, anti-EGFR strategies are used as anti-cancer agents^2^. Recent clinical reports, however, have disappointingly shown that, even though gefitinib has indicated some anti-tumor action against CRC, a high level of novel resistance has occurred in response to such treatment^3^,^4^. Therefore, many new biomarkers have been identified that can potentially predict the response of CRC patients to gefitinib.

Signal transducer and activator of transcription 3 (STAT3) is a member of the STAT family of transcription factors, and is activated in several cancers^5^. STAT3 tyrosine phosphorylation can be stimulated by the activation of the upstream receptor and/or non-receptor kinases including EGFR, IL-6, and Janus-activated kinases (JAK), and Src family kinases^6^,^7^,^8^. STAT3 activation has been associated with resistance to EGFR-TKI in preclinical models of glioma and head and neck squamous cell carcinoma (HNSCC)^5^,^9^. And resistance in patients who have non-small cell lung cancer (NSCLC) to neoadjuvant EGFR-TKI therapy is associated with elevated STAT3 activity in tumors^10^. These cumulative results suggest that targeting STAT3 may overcome the resistance to EGFR-TKI in cancer cells. However, STAT3 is not an ideal molecular target for CRC therapy given the potential damage to normal tissue and other off-target effects. Gao *et al.* showed that nuclear pyruvate kinase isoform M2 (PKM2) regulates that constitutive activation of STAT3 in CRC cells^11^. If nuclear PKM2 is expressed differentially in gefitinib-resistant CRC cells as opposed to gefitinib-sensitive CRC cells, nuclear PKM2 may be an ideal target for treatment with gefitinib.

Pyruvate kinase (PK) acts as a rate-limiting enzyme in the last step of the glycolytic pathway. This pathway catalyzes phosphoenolpyruvate (PEP) conversion to pyruvate, which is achieved by the transfer of a phosphate from PEP to ADP^12^. Mammals have four PK isoforms (L, R, M1, and M2), and the liver and red blood cells are the sites of L and R isoform expression. Most adult tissues of mammals express the M1 isoform, while the M2 isoform, which is a variant resulting from M1 splicing, is expressed in embryonic and tumor tissues^13^. The catalytically active PKM2 is a tetramer that interacts with a glycolytic enzyme complex^14^. In tumor cells, PKM2 becomes a dimer and seems to be catalytically unable to convert PEP to pyruvate^15^. It has been suggested that inactive PKM2 assists with tumor progression because it channels the carbon source from glycolytic intermediates to biosynthesis. This especially affects the synthesis of lipids, nucleic acids and proteins, which are required for cell proliferation^11^. Recently, several independent reports have indicated that PKM2 localizes to the cell nucleus in response to various signals^16^,^17^. Nuclear PKM2 participates in the regulation of gene transcription of targets, such as OCT-4, HIF-1α, cyclin D1 and c-Myc^18^,^19^,^20^. In addition, the inhibition of PKM2 by RNA interference sensitizes gastric carcinoma and NSCLC cells to cytotoxic drugs^21^,^22^. However, it is not clear whether nuclear PKM2-induced STAT3 phosphorylation has a significant role in the regulation of gefitinib sensitivity in CRC.

In our study, we show that nuclear PKM2 protein levels correlate with gefitinib resistance in CRC cells, which is mediated by the STAT3 pathway. The growth of gefitinib-resistant CRC cells *in vivo* and *in vitro* was inhibited by co-targeting EGFR and STAT3 phosphorylation. These observations indicate that nuclear PKM2 is a possible molecular target for sensitizing CRC cells to EGFR-TKI therapy.

## Results

### Nuclear PKM2 protein levels correlate with gefitinib resistance in CRC cells

**Figure 1:**
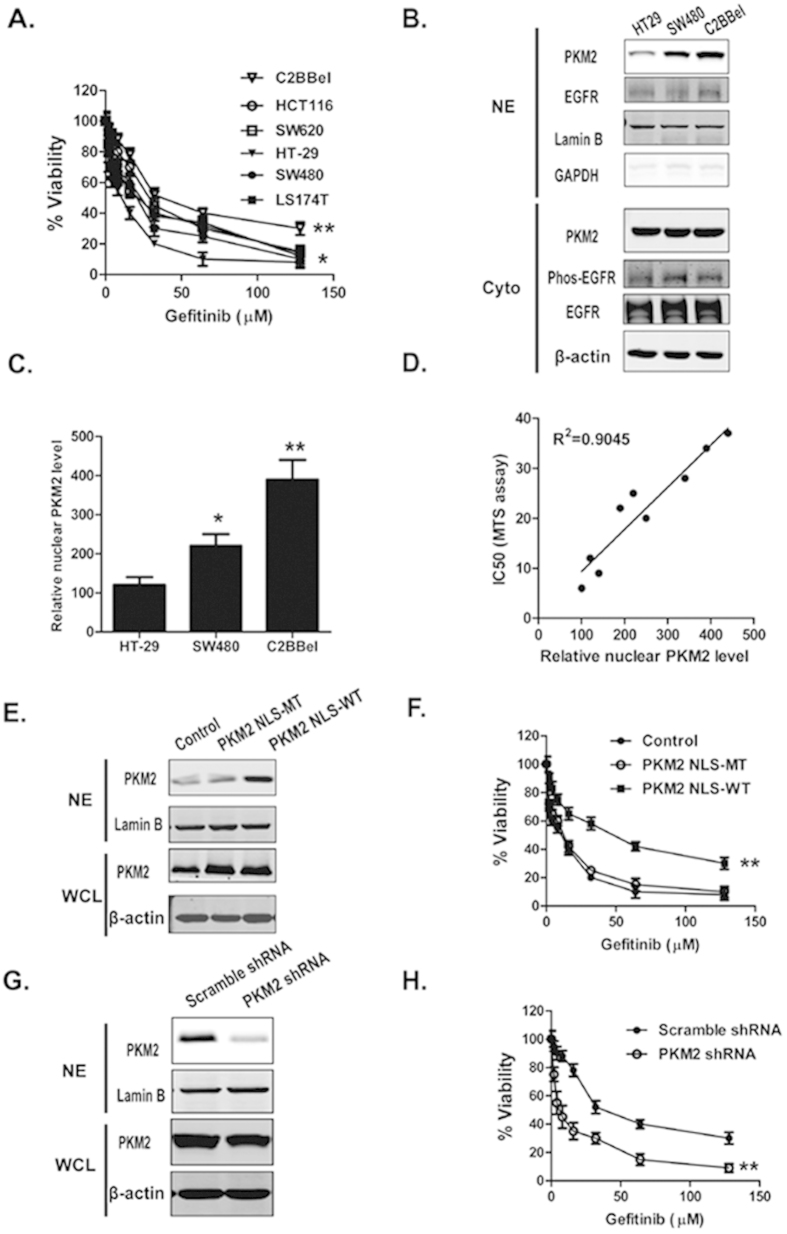
Nuclear PKM2 expression in CRC cell lines correlates with their resistance to gefitinib. (**A**) Representative CRC cell lines were treated with varying concentrations of gefitinib. MTS assays were conducted, and IC50 values were calculated after 72 h (^*^P < 0.05, ^**^P < 0.01). (**B**) The expression of PKM2, phosphorylation and total EGFR were detected in nuclear extracts (NE) and cytoplasmic extracts (Cyto) of three representative CRC cell lines using Western blot analysis. Lamin B or β-actin were loading controls. GAPDH in nuclear extracts was analyzed as a control showing there were no cytoplasmic protein contaminations. (**C**) The protein levels of nuclear PKM2 were quantified by densitometry analysis, and the reading was normalized to Lamin B. The error bars represent the S.D. of the mean values of three experiments. A Student’s *t*-test was used to assess the significance of the differences in the nuclear PKM2 between the cells lines. (**D**) The association between nuclear PKM2 protein expression levels and gefitinib sensitivity of CRC cells was evaluated using Pearson analysis. The coefficient of determination (R^2^) in the linear regression model was calculated. (**E**,**G**) The expression of PKM2 in HT29 (**E**) and C2BBel (**G**) cells were analyzed by immunoblot of nuclear extracts (NE) and whole-cell lysates (WCL) of cells in which nuclear PKM2 was overexpressed or knocked down. (**F**,**H**) The viability was analyzed using the MTS assays in HT29 (**F**) and C2BBel (**H**) cells treated with different concentrations of gefitinib. Nuclear PKM2 was overexpressed or knocked down in HT29 (**F**) and C2BBel (**H**) cells, respectively.

To understand whether nuclear PKM2 was a possible target for gefitinib resistance, six CRC cell lines, HT29, SW480, SW620, LS174T, HCT116 and C2BBel, were employed to evaluate a possible correlation between gefitinib resistance and nuclear PKM2 expression levels. The viability of cancer cells after gefitinib treatment was determined using the MTS assay. HT29, SW480 and C2BBel were three representative CRC cell lines with significant differences in their sensitivity to gefitinib and were chosen from the CRC cells lines for subsequent studies (Fig. 1A). PKM2 expression levels were determined in the three cell lines using Western blot analysis. The increased nuclear PKM2 levels were not due to variations in PKM2, phospho-EGFR, and total EGFR expression in cytoplasmic extracts (Fig. 1B). The levels of nuclear PKM2 were analyzed with densitometry (Image J software, Bethesda, MD, USA). The data were normalized to lamin B. Semi-quantification of the immunoblots indicated that nuclear PKM2 was expressed at the highest levels in C2BBel cells and the next highest levels were in SW480 and HT29 cells (Fig. 1C). The levels of nuclear PKM2 were correlated with IC50 of CRC cells that received gefitinib treatment, with a correlation coefficient (R^2^) of 0.9045 (Fig. 1D).

The nuclear PKM2 protein levels were overexpressed in HT29 cells, or were knocked down in C2BBel cells using vector-medicated transfection to verify the role of nuclear PKM2 in gefitinib resistance (Fig. 1E,G). The transfection efficiency was also assessed using an immunofluorescence assay (Supplementary Fig. S1). The overexpression of nuclear PKM2 rendered HT29 cells more resistant to gefitinib compared with the PKM2 vector with nuclear localization sequence (NLS) mutation-transfected cells. Reciprocally, the shRNA knockdown of PKM2 had the effect of significantly increasing the sensitivity of C2BBel cells when treated with gefitinib in relation to nonspecific shRNA-transfected cells (Fig. 1F,H). These observations demonstrated that nuclear PKM2 regulated gefitinib resistance and suggested that nuclear PKM2 was a possible molecular target for gefitinib sensitivity in CRC.

### Nuclear PKM2 increases resistance to apoptosis and cell cycle arrest induced by gefitinib

**Figure 2:**
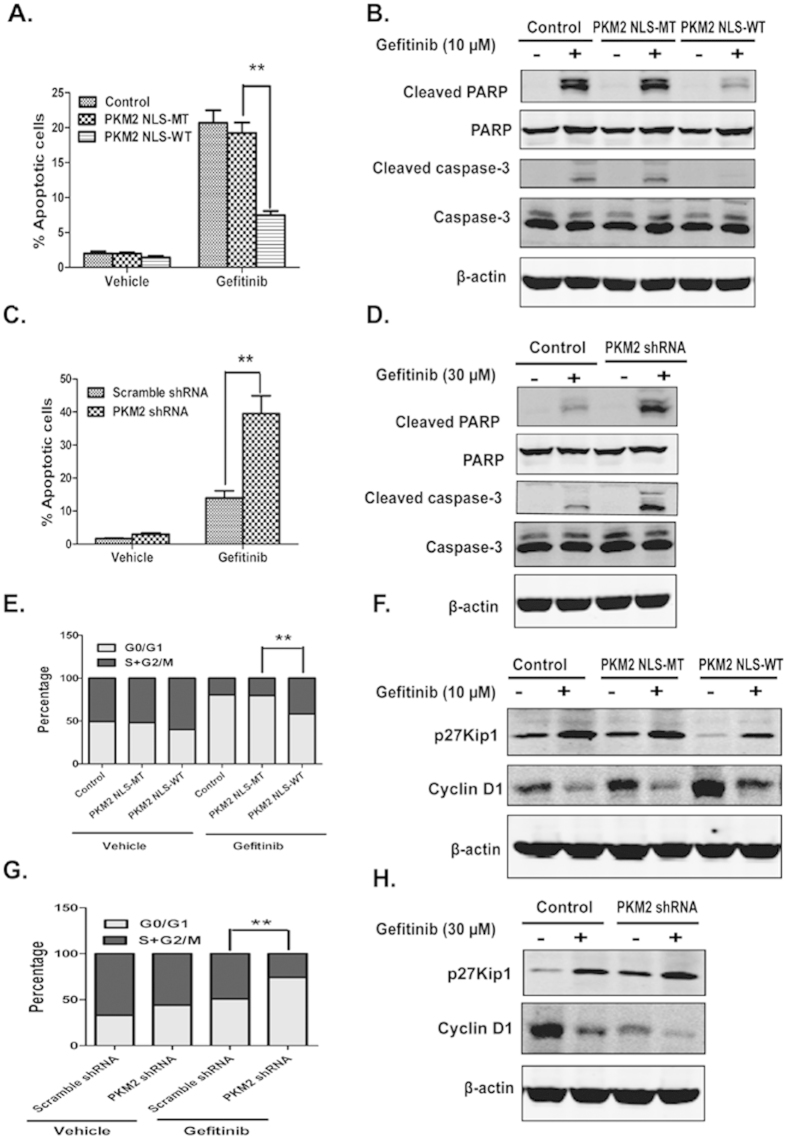
The overexpression of nuclear PKM2 suppresses the apoptosis and cell cycle arrest of CRC cells induced by gefitinib. (**A,C**) Flow cytometry was used to analyze the annexin V-positive population of HT29 or C2BBel cells. The final concentration of gefitinib was 10 μM or 30 μM. (**B**,**D**) Cleaved PARP and caspase-3 were detected with Western blotting in HT29 or C2BBel cells, which were transfected with PKM2-NLS or PKM2 shRNA. (**E**,**G**) Flow cytometry was used to detect the proportions of cells in G1, S and G2-M phases. The final concentration of gefitinib was 10 μM or 30 μM. (**F**,**H**) Cyclin D1 and p27Kip1 were detected with Western blotting in HT29 or C2BBel cells, which were transfected with PKM2-NLS or PKM2 shRNA.

However, the mechanism by which nuclear PKM2 regulates gefitinib resistance in CRC requires further investigation. To determine the biological significance of nuclear PKM2 in CRC, gefitinib-induced apoptosis was initially analyzed in HT29 cells overexpressing nuclear PKM2. The tumor cells infected with mutant-NLS vector were used as a control. Analysis with flow cytometry indicated that gefitinib significantly caused the turnover of annexin V, an indicator of apoptosis; overexpression of nuclear PKM2 lowered the percentage of annexin V-positive cells in the HT29 cell line (Fig. 2A and Supplementary Fig. S2A). Analysis with Western blotting indicated that gefitinib radically induced PARP and caspase-3 cleavage, which are markers of apoptosis; while overexpression of nuclear PKM2 attenuated the gefitinib-induced cleaved caspase-3 and PARP (Fig. 2B).

As confirmation of the effects of nuclear PKM2 on CRC cell apoptosis, gefitinib-induced apoptosis was analyzed in C2BBel cells transfected with the PKM2 shRNA vector, and the tumor cells transfected with nonspecific shRNA acted as controls. Analysis with flow cytometry indicated that transfection of the PKM2 shRNA vector induced an increase in the percentage of annexin V-positive C2BBel cells treated with gefitinib (Fig. 2C and Supplementary Fig. S2B). Furthermore, Analysis with Western blotting indicated that PKM2 knockdown enhanced the levels of cleaved PARP and caspase-3 induced by gefitinib treatment (Fig. 2D). These observations suggested that nuclear PKM2 attenuated gefitinib-induced apoptosis.

We next investigated the effects of nuclear PKM2 on cell cycle after staining the cells with DNA-intercalating dye, propidium iodide. The sensitivity of CRC cells to gefitinib may be due to the arrest of the cell cycle at G1 phase. Figure 2E,G showed that nuclear PKM2 overexpression decreased the number of gefitinib-treated HT29 cells in the G1 phase. Moreover, PKM2 knockdown significantly changed the distribution of cell cycle phases in gefitinib-treated C2BBel cells (Supplementary Fig. S3A,B). We next examined the effects of PKM2 on protein levels of cyclin D1 and p27Kip1, both of which are cell cycle regulatory molecules. Analysis with Western blotting indicated that nuclear PKM2 overexpression attenuated the change in cyclin D1 and p27Kip1 in HT29 cells treated with gefitinib. Cyclin D1 was significantly downregulated after the combined treatment with gefitinib and PKM2 shRNA, whereas the cell cycle inhibitor p27Kip1 was upregulated (Fig. 2F,H). Taken together, these data showed that nuclear PKM2 inhibited gefitinib-induced cell cycle arrest and that combined treatment with gefitinib and PKM2 shRNA significantly facilitated the cell cycle arrest of gefitinib-resistant C2BBel cells.

### Phospho-STAT3 levels correlate with gefitinib resistance in CRC cells and are regulated by nuclear PKM2

**Figure 3:**
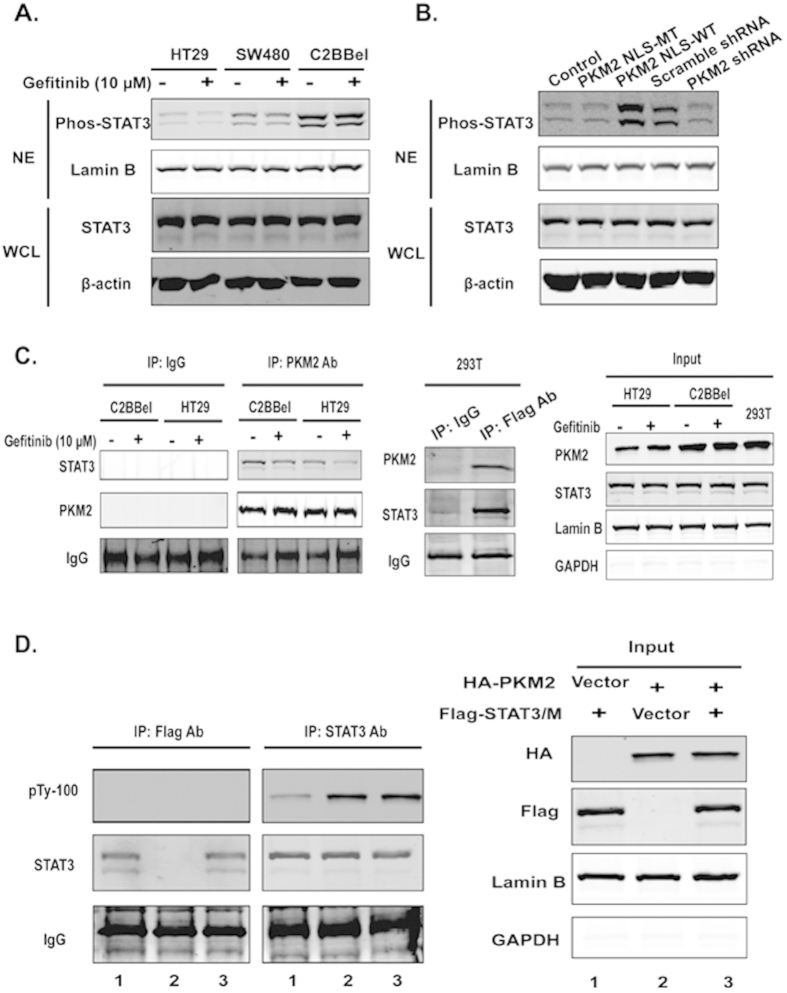
Nuclear PKM2-induced STAT3 phosphorylation correlates with gefitinib resistance in CRC cells. (**A**) Representative CRC cell lines were treated with gefitnib or left untreated. At 48 h after treatment, the cells were lysed and subjected to immunoblot analysis. The levels of phosphorylated and total STAT3 in nuclear extracts (NE) and whole-cell lysates (WCL) were detected. (**B**) Total and phosphorylated STAT3 were detected with Western blotting in HT29 or C2BBel cells, which were transfected with PKM2-NLS or PKM2 shRNA, respectively. (**C**) STAT3 phosphorylation was regulated by nuclear PKM2 through physical binding. The left panel shows Western blotting results of STAT3 after PKM2 immunoprecipitation (IP); the middle and right panel shows Western blot results for the reciprocal IP and loading control. (**D**) Phosphorylation of endogenous STAT3 (IP: STAT3) or exogenously expressed mutant Y705A (IP: Flag) by PKM2 in SW480 cell nucleus was analyzed by immunoprecipitation using anti-STAT3 antibody (IP: STAT3) or anti-Flag antibody (IP: Flag) followed by immunoblot using antibody against phospho-tyrosine (IB: pTy-100).

STAT3 phosphorylation has been implicated in gefitinib resistance in human HNSCC and NSCLC^5^,^10^. We therefore investigated whether STAT3 was activated by nuclear PKM2 and was involved in gefitinib resistance in CRC. To determine whether STAT3 phosphorylation had a dose-response relationship with gefitinib resistance in CRC cells, the three CRC cell lines, including HT29, SW480 and C2BBel, were chosen for the experiments. STAT3 phosphorylation in the three cell lines was measured with or without gefitinib treatment. Similar to the nuclear PKM2 protein levels, STAT3 phosphorylation highly correlated with gefitinib sensitivity in CRC cells (Fig. 3A).

To determine the mechanism of regulation by nuclear PKM2 on STAT3 activation in CRC cells, the effects of nuclear PKM2 on STAT3 activation/phosphorylation were investigated in CRC cells in which nuclear PKM2 was either knocked down or overexpressed. First, our data showed that upregulation of STAT3 phosphorylation occurred in nuclear PKM2-transfected cells when compared with the controls; however, STAT3 phosphorylation was downregulated in C2BBel cells with PKM2 shRNA knockdown (Fig. 3B). Furthermore, co-immunoprecipitation assays showed that PKM2 bound to STAT3, but when the cells were treated with gefitinib, this association decreased, which may be due to the decrease in STAT3 activation. Finally, reciprocal immunoprecipitation using Flag antibodies pulldown of STAT3 and subsequent probe of immunocomplexes with PKM2 suggested that STAT3 was bound to PKM2 in the nuclear extracts of 293T cells (Fig. 3C).

We created a Flag-tagged STAT3 mutant (Y705A) and HA-NLS-PKM2 in SW480 cells to determine whether the Y705 residue of STAT3 was the single phosphorylation site for PKM2. Activation of exogenously and endogenously expressed STAT3 was analyzed with immunoprecipitation of Flag-tagged STAT3 mutants or endogenous STAT3. This was followed by immunoblotting with an antibody against phosphotyrosine. The results showed that the endogenous STAT3 was phosphorylated, whereas the exogenously expressed mutant did not show any phosphorylation (Fig. 3D), which suggested that Y705 was the single activation site for PKM2 in the nuclear extracts.

### Nuclear PKM2-induced gefitinib resistance is STAT3 dependent

**Figure 4:**
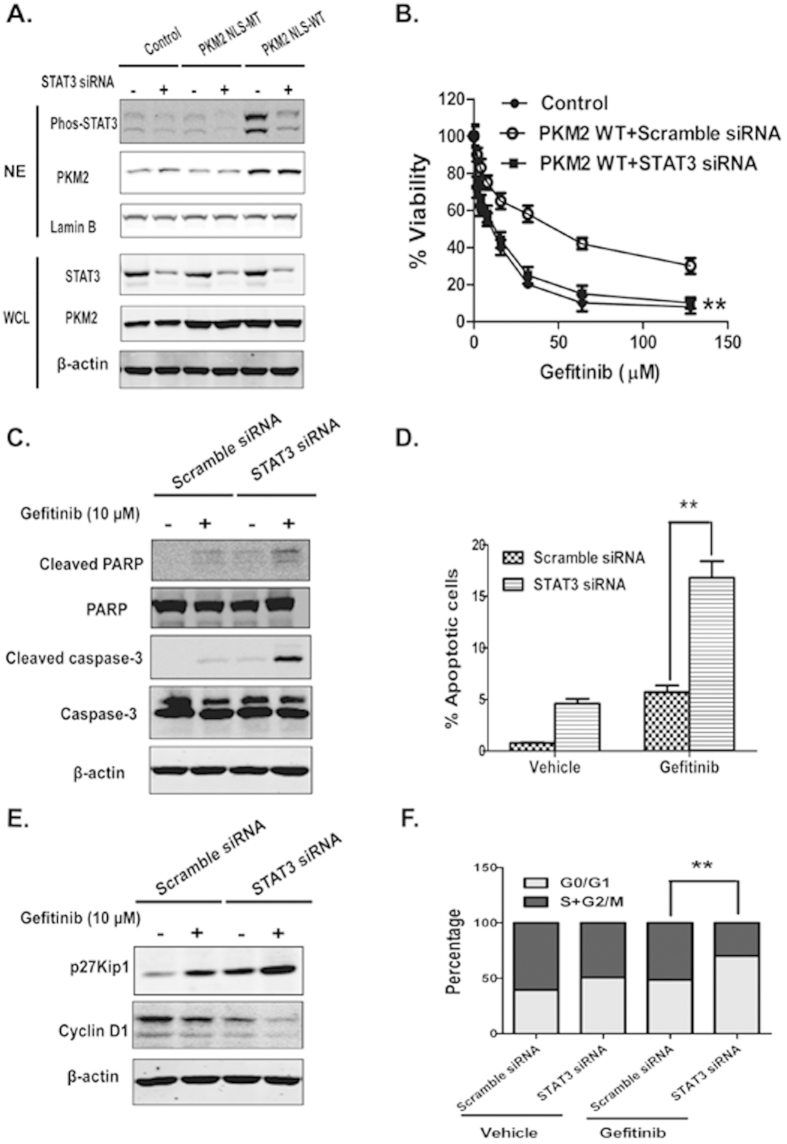
Nuclear PKM2-induced gefitinib resistance is mediated by STAT3. (**A**) STAT3 was knocked down in HT29/PKM2-NLS cells by siRNA (50 nM final concentration for both STAT3-specific and negative control siRNA). (**B**) STAT3 siRNA reinstated gefitinib sensitivity of HT29/PKM2-NLS cells. The indicated cells lines were treated with gefitinib for three days, which was followed by an MTS assay, at two days after transfection. (**C**) Cleaved PARP and caspase-3 were detected with Western blotting in HT29/PKM2-NLS cells, which were transfected with STAT3 siRNA. (**D**) Flow cytometry was used to analyze the annexin V-positive population of HT29/PKM2-NLS cells. The final concentration of gefitinib was 10 μM. (**E**) Western blot analysis was employed to detect the expression level of cyclin D1 and p27kip1 in HT29/PKM2-NLS cells, which were transfected with STAT3 siRNA. (**F**) The proportions of cells in the different phases were quantified using flow cytometry. The final concentration of gefitinib was 10 μM.

STAT3 was inhibited in HT29 cells using siRNA or the small molecule inhibitor Stattic to confirm the role of STAT3 in nuclear PKM2-induced gefitinib resistance. Successful inhibition of STAT3 expression and phosphorylation was confirmed using Western blot analysis (Fig. 4A and Supplementary Fig. S4). Gefitinib sensitivity was restored in nuclear PKM2-overexpressed cells after siRNA knockdown of STAT3, according to an MTS assay, suggesting that nuclear PKM2-induced gefitinib resistance was dependent on STAT3 (Fig. 4B). STAT3 depletion in HT29/PKM2-NLS cells led to an increase in cleavage caspase-3 and PARP induced by gefitinib; this event was accompanied by an increase in annexin V-positive cells in HT29/PKM2-NLS cells treated with gefitinib (Fig. 4C,D and Supplementary Fig. S2C). As shown in Fig. 4E,F, STAT3 knockdown significantly changed the cell cycle arrest in HT29/PKM2-NLS cells treated with gefitinib (See Supplementary Fig. S3C). STAT3 siRNA led to the downregulation of cyclin D1 and increased p27Kip1 expression after gefitinib treatment. This result was consistent with earlier data and showed that nuclear PKM2 modulated gefitinib resistance via a STAT3-dependent process.

### Nuclear PKM2 increases CRC resistance to gefitinib *in vivo*

**Figure 5:**
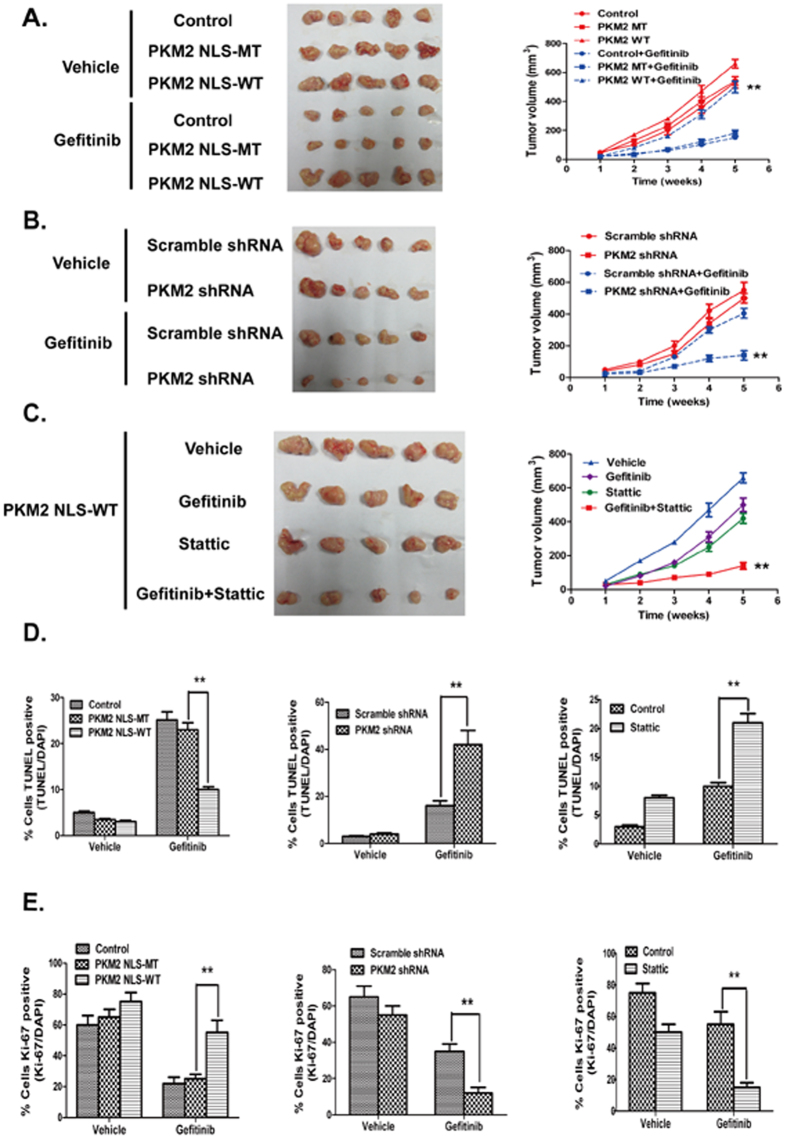
Nuclear PKM2 decreases the sensitivity of CRC cells to gefitinib *in vivo*. (**A**,**B**) The tumor images from the nuclear PKM2 overexpression, PKM2 knockdown, or the control group were determined at the 28th day with or without gefitinib treatment (50 mg/kg/day), and the tumor growth rate were analyzed between the groups. (**C**) Stattic sensitized HT29/PKM2-NLS cells to gefitinib *in vivo*. Mice were subcutaneously implanted with HT29/PKM2-NLS cell lines. Mice were administrated with vehicle (saline), Stattic at 10 mg/kg/day, gefitinib at 50 mg/kg/day, or the combination of the 2 drugs for 4 weeks. (**D**) The TUNEL-positive cells were analyzed in the tumor sections. We counted the number of nuclei that were positively stained to determine the percentage of TUNEL-positive cells in the groups, using three randomly selected fields in each slide. (**E**) The Ki67-positive cells were analyzed in the tumor sections; the percentage of Ki67-positive cells in the groups was counted.

The impact of nuclear PKM2 on CRC resistance to gefitinib was evaluated *in vivo*, in which Balb/c nude mice were employed as a xenograft tumor model. The HT29 cells that overexpressed nuclear PKM2 were injected subcutaneously into the bilateral axilla of nude mice. After 7 days, when the tumors were clearly palpable, gefitinib was administered at a dose of 50 mg/kg/day by oral gavage. As shown in Fig. 5A, gefitinib showed no effect of inhibition on the volume of nuclear PKM2-transfected HT29 xenograft tumors. In addition, with gefitinib treatment, the tumors grew at a higher rate in the group showing nuclear PKM2 overexpression than in group used as controls, which suggested that nuclear PKM2 augmented CRC resistance to gefitinib treatment. We tested the effect of nuclear PKM2 in C2BBel cells in a mouse xenograft model to verify these findings. Not surprisingly, PKM2 knockdown inhibited the growth of gefitinib-resistant C2BBel xenograft tumors significantly (Fig. 5B). Analysis using *in situ* terminal deoxynucleotidyl transferase dUTP nick-end labeling (TUNEL) with cryogenic sections from xenograft tumors that overexpressed nuclear PKM2 exhibited fewer cells that were TUNEL-positive compared to controls, whereas PKM2 knockdown increased the proportion of apoptotic cells (Fig. 5D and Supplementary Fig. S5). Anti-Ki67 histological analysis showed that the nuclear PKM2-overexpressing tumors had substantially higher rates of proliferation compared to the PKM2-NLS-mutant tumors after gefitinib treatment. Moreover, PKM2 knockdown significantly inhibited the proliferation of gefitinib-resistant C2BBel xenograft tumors (Fig. 5E and Supplementary Fig. S6). In addition, experiments using xenografts and HT29/PKM2-NLS cells indicated that the Stattic and gefitinib combination therapy reversed nuclear PKM2-induced gefitinib resistance and retarded tumor growth compared to either single drug administration (Fig. 5C). This synergistic effect was likely due to the increased number of cells undergoing apoptosis and decreased number of proliferating cells, as measured by the TUNEL and IF assays, respectively (Fig. 5D,E, Supplementary Figs S5 and S6).

## Discussion

**Figure 6:**
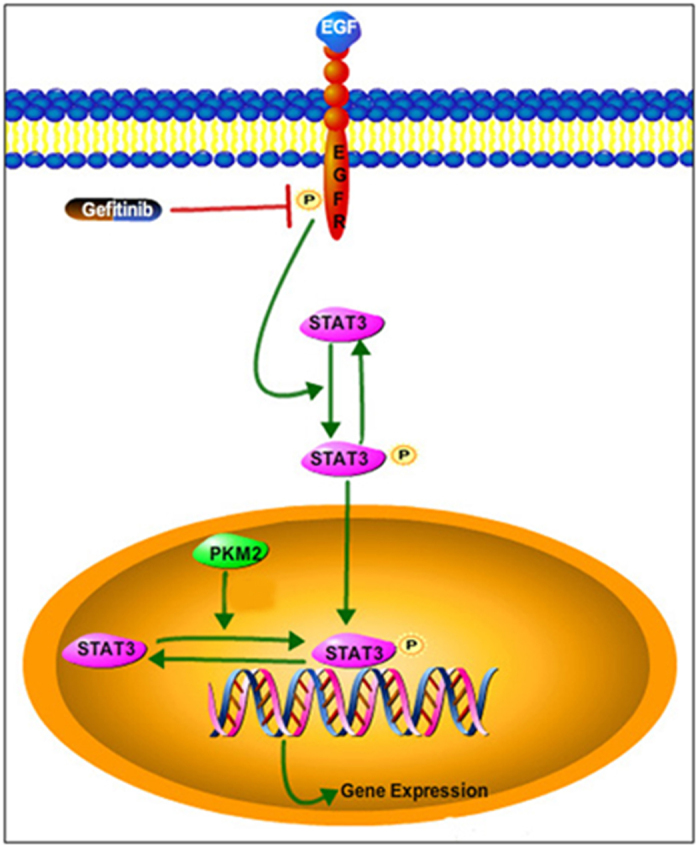
Schema illustrating the mechanism of nuclear PKM2-regulated resistance to gefitinib in CRC. This figure was drawn by Q.L.

Despite the anti-tumor effects of anti-EGFR-based therapies observed in many studies, gefitinib is clinically inactive in metastatic CRC^23^. Several studies have indicated that wild-type KRAS, BRAF, PIK3CA, and phosphatase and tensin homolog (PTEN) proteins predict resistance to cetuximab as examined in a panel of CRC cell lines. However, these biomarkers may not contribute to gefitinib resistance in CRC^24^. The underlying molecular mechanisms for resistance to gefitinib treatment are not currently known. The high cost of treatment and toxicity associated with gefinitib necessitates deeper understanding of the molecular and/or clinical predictors of responses to treatment, so that better selection of patients for treatment can be made^23^. Herein, we determine that increased nuclear PKM2 expression in CRC is one of the underlying mechanisms for intrinsic resistance to EGFR-TKIs. The results of our study suggest the following model: the expression of nuclear PKM2 induces the initiation of STAT3 signalling, which then offsets the impact of EGFR-TKI on cell apoptosis and proliferation (Fig. 6). The model we present here suggests that targeting both STAT3 and EGFR activation may be effective strategy to overcome nuclear PKM2-induced resistance to EGFR-TKIs in CRC patients.

Nuclear PKM2 levels were inversely correlated with gefitinib sensitivity in CRC cells, which supported the idea that high nuclear PKM2 expression causes gefitinib resistance. Consistent with this result, we found that the vector-mediated nuclear PKM2 overexpression in HT29 cells affected gefitinib treatment effects and increased tumor volumes compared with controls in the xenograft mice model. In contrast, PKM2 knockdown had the effect of improving gefitinib treatment and inhibited tumor growth rate. As numerous reports have shown, EGFR subcellular distribution into the nucleus might play a role in cetuximab therapy resistance^25^,^26^. However, there was no relationship between the amount of nuclear EGFR and gefitinib resistance in the CRC cells. In our study, we hypothesized that nuclear PKM2-induced proliferation and survival of cancer cells affected the anticancer efficacy of gefitinib in CRC, and performed experiments to test this hypothesis. Our results clearly indicated that nuclear PKM2 overexpression in HT29 cells attenuated the cell death and G1 arrest effects of gefitinib. As shown in Figs 2 and 5, gefitinib-induced apoptosis and cell cycle arrest were increased substantially by PKM2 reduction, as analysed by flow cytometry and apoptotic and cell cycle arrest markers. These results indicate that the p27Kip1 and caspase-3 pathways regulated by nuclear PKM2 are important in gefitinib-induced cell growth inhibition and apoptosis. Further studies are necessary to determine the underlying mechanisms for nuclear PKM2 regulation of gefitinib resistance.

The STAT3 pathway is activated in many cancer types and is related with oncogenic transformation, angiogenesis, invasion, and metastasis and immune system suppression. Several studies have implicated STAT3 activation in EGFR resistance^27^,^28^. In hepatocellular carcinoma, cetuximab resistance is mediated via STAT3 activation, and therapies that combined inhibitors of STAT3 and EGFR exhibits enhanced growth inhibition *in vitro*^29^. In NSCLCs, STAT3 activation confers resistance against gefitinib, suggesting that, in patients with NSCLCs who are not sensitive to EGFR inhibitors, STAT3 targeting may be an alternative therapy^30^. In high-grade glioma, elevated pSTAT3 levels have been linked to chemo-resistance, and blockade of STAT3 signalling sensitizes glioma cells to chemotherapy, thus providing a rationale for the use of targeted therapies against STAT3^9^. The previous study show that nuclear PKM2 enhances the proliferation of tumor cells by phosphorylating STAT3 at Y705^11^. In addition, nuclear PKM2-dependent β-catenin transactivation is necessary for EGF-promoted gene expression, cell proliferation and tumorigenesis^20^. However, the importance of nuclear PKM2-induced STAT3 phosphorylation in the regulation of gefitinib sensitivity has been unknown. Our observations indicate that nuclear PKM2 regulated gefitinib resistance in CRC cells by altering STAT3 activity instead of increasing STAT3 protein expression *in vitro* and *in vivo*. Moreover, PKM2 is physically bound to STAT3 in the nucleus, and STAT3 Y705 is the only PKM2 site of phosphorylation in the nucleus. Similar to the *in vitro* results indicating that inhibition of STAT3 sensitized HT29/PKM2-NLS cells to gefitinib, Stattic and gefitinib combination therapy significantly decreased the growth HT29/PKM2-NLS xenograft tumors, laying the basis for combination therapy as the rational treatment for this class of CRCs.

Our study creates new avenues for overcoming gefitinib resistance by showing that molecules that inhibit the nuclear functions of PKM2 or disrupt STAT3 association with PKM2 in the nucleus may increase the sensitivity of CRC cells to gefitinib. However, the safety of translocation inhibitors of nuclear PKM2 or molecules that disrupt PKM2/STAT3 association has not yet been evaluated. The Ras/Raf/ERK pathway is a major signalling pathway that is involved in gefitinib resistance and cell proliferation^31^. The Lu group reported that ERK1/2 promotes the translocation of PKM2 to the nucleus in glioblastoma multiforme cells through phosphorylation at S37^32^. It remains unknown whether ERK1/2 co-functions with nuclear PKM2 to modulate EGFR-TKI resistance in CRC cells.

In summary, we show that nuclear PKM2 overexpression could represent a new mechanism for intrinsic EGFR-TKI resistance in CRC patients. Our results indicate that co-targeting of STAT3 and EGFR activation may be an ideal approach for the treatment of gefitinib-resistant, nuclear PKM2-positive CRC patients. Moreover, since STAT3 mediates nuclear PKM2’s control of gefitinib resistance, small molecules that disrupt the interaction of nuclear PKM2 with STAT3 might enhance gefitinib-induced growth inhibition and apoptosis and may therefore be helpful in increasing the efficacy of EGFR-TKI therapy.

## Methods

### Cell lines and reagents

The HT29, SW480, SW620, LS174T, HCT116, C2BBel and HEK293T cell lines were purchased from ATCC (American Type Culture Collection, Manassas, VA, USA). HT29, SW480, HCT116 and C2BBel cells were maintained in RPMI-1640 medium (Invitrogen, Carlsbad, CA, USA) supplemented with 10% heat-inactivated foetal bovine serum (FBS, Invitrogen); LS174T and HEK293T were maintained in Dulbecco’s modified Eagle’s medium (DMEM, Invitrogen) supplemented with 10% FBS, and SW480 cells were maintained in L15 medium (Invitrogen) supplemented with 10% FBS. The cells were incubated at 37 °C with 5% CO_2_. The following commercial antibodies were obtained from Cell Signaling (Danvers, MA, USA): rabbit monoclonal to PKM2; phospho-STAT3 (Y705); cleaved PARP; cleaved caspase-3; cyclin D1; p27Kip1; and mouse monoclonal to STAT3. The secondary antibodies used in the experiments were IRDye^®^ 680 donkey anti-mouse IgG, IRDye^®^ 800CW goat anti-rabbit IgG (LI-COR Biosciences, Lincoln, NE, USA), and Alexa Fluor 594 goat anti-rabbit IgG (Invitrogen). Gefitinib and STAT3 inhibitor V (Stattic) were purchased from Santa Cruz Biotechnology (Santa Cruz, CA, USA).

### MTS assay

Cells were seeded in 96-well plates at a density of 2,000 cells per well overnight in medium with 10% FBS. Cells were then treated with the relevant reagents for 3 days. We determined the number of viable cells with an MTS assay kit (Promega, Madison, WI, USA) according to the manufacturer’s protocol. Each assay comprised triplicates and each assay was independently repeated at least three times. The data are shown as the percentage of control cells calculated from the absorbance corrected for background.

### Vector construction and transfection

To construct the wild-type and alanine-substituted mutant of NLS-tagged vector, the full-length human PKM2 gene fused to NLS tag (RRRHIVRKRTLRR) was synthesized and cloned in-frame into pLenti6.3-MCS-IRES2-EGFP vector (Invitrogen) that had been digested with NheI and AscI. The wild-type and phospho-Y705 mutant of Flag-tagged STAT3 vector was constructed as described above. To construct the PKM2 shRNA vector, oligonucleotides were synthesized (sense: 5- CACCGCTGTGGCTCTAGACACTAAACGAATTTAGTGTCTAGAGCCACAGC-3, antisense: 5-AAAAGCTGTGGCTCTAGACACTAAATTCGTTTAGTGTCTAGAGCCACAGC-3) and cloned into the pENTR/U6-GFP vector (Invitrogen) after annealing. This product was then recombined via the LR reaction and subcloned into the pLenti6/Block-it-DEST vector (Invitrogen). To achieve stable transfection, the transfected cells were selected with antibiotic blasticidin (Invitrogen).

### Western blotting

Nuclear protein extract was prepared using subcellular protein fractionation kit (Thermo Scientific, Rockford, IL, USA). SDS-polyacrylamide gels of 8–12% were used for protein resolution. Electroblotting to nitrocellulose membranes (Bio-Rad, Hercules, CA, USA) was then performed. Detection of proteins of interest was performed with specific antibodies, followed by IRDye^®^ 680 or IRDye^®^ 800-conjugated secondary antibodies. An Odyssey infrared imaging system (LI-COR Biosciences) was used to scan the blots.

### Immunoprecipitation

Nuclear protein extracts were pre-cleared with protein A/G plus beads (Santa Cruz Biotechnology). A total of 500 μg of protein from each sample was incubated with the indicated antibodies at 4 °C overnight at a dilution of 1:100. The protein samples were further incubated with 15 μl of pre-cleared protein A/G plus beads for 2 h at 4 °C, followed by 3 washes with non-denaturing lysis buffer. The prepared samples were then detected with Western blot assays, as described above.

### Apoptosis assay

The cells were harvested and resuspended in 1× binding buffer at a concentration of 1 × 10^6^ cells/ml. Then, 100 μl of cell suspension were stained with 5 μl of annexin V for 15 min at room temperature in the dark. After staining, 10 μl of propidium iodide and 400 μl of binding buffer were added to the cell suspension, and the mixture was analyzed using a FACSCalibur flow cytometer (BD, Franklin Lakes, NJ, USA).

### Cell cycle analysis

Approximately 1 × 10^6^ cells were collected and then fixed with 75% cold ethanol at 4 °C overnight. DNA staining was performed by adding 500 μl of propidium iodide/RNase (the final concentration of propidium iodide: 50 μg/ml) to the cells. The percentage of cells in each cell cycle phase was measured by FACStar Plus dual laser system and a FACSort system (BD).

### TUNEL and fluorescent immunostaining analysis

OCT-embedded sections of tumors were fixed with 4% paraformaldehyde. The slides were stained with TUNEL reaction mixture or primary antibodies after permeabilization with 0.1% Triton X-100 in sodium citrate for 10 min. One drop of Vectashield Mounting Medium with DAPI (Vector Laboratories, Burlingame, CA, USA) was added to each slide before photography. The slides were observed under a microscope (Leica DM2500, Germany).

### Xenograft tumor model

Four-week-old BALB/c nude mice were purchased from Shanghai Laboratory Animal Center (Shanghai, China). All animal work was approved by Ethical Review Board of the Medical Faculty of Renji Hospital and was handled according to the guidelines approved by this committee. A total of 5 × 10^6^ tumor cells that either overexpressed PKM2 or with PKM2 knockdown were injected subcutaneously into the flanks of athymic nude mice. When the tumor volumes reached approximately 30–50 mm^3^, 5 mice from each group were administered a daily oral dose of gefitinib at 50 mg/kg/day for 4 weeks. Tumor volume (V) was measured once per week using the formula: V = 1/2 (L × W^2^), where L is the length, and W is the width. In the combination treatment experiments, the mice were divided into 4 groups after the tumors were approximately 30–50 mm^3^, and were treated with the following: (a) saline (vehicle) daily for 4 weeks; (b) gefitinib at 50 mg/kg/day for 4 weeks; (c) Stattic at 10 mg/kg/day for 4 weeks; and (d) gefitinib at 50 mg/kg and Stattic at 10 mg/kg daily for 4 weeks. After 4 weeks, the mice were euthanized, and the tumors were excised.

### Statistical analysis

Data are shown as the mean ± standard deviation (S.D.) or standard error (S.E.M.). All categorical data were compared with a Fisher’s exact test or X^2^ test. The correlation between nuclear PKM2 protein levels and gefitinib IC50 was analyzed using Pearson analysis. A Student’s *t* test or Mann-Whitney U-test was used when appropriate. All tests were two-sided, and *P *< 0.05 was considered significant.

## Additional Information

**How to cite this article**: Li, Q. *et al.* Nuclear PKM2 contributes to gefitinib resistance via upregulation of STAT3 activation in colorectal cancer. *Sci. Rep.* **5**, 16082; doi: 10.1038/srep16082 (2015).

## Supplementary Material

Supplementary Information
