# Supplementary material for: Nuclear PKM2 contributes to gefitinib resistance via upregulation of STAT3 activation in colorectal cancer
Source: Sci Rep. 2015 Nov 6;5:16082. doi: 10.1038/srep16082 (PMC4635355; doi:10.1038/srep16082)
Supplement: Supplementary Information [file srep16082-s1.pdf]

Nuclear PKM2 contributes to gefitinib resistance via upregulation of STAT3 activation in colorectal cancer

Qiong Li[1], Daoxiang Zhang[2], Xiaoying Chen[1], Lei He[1], Tianming Li[1], Xiaoping Xu[1], Min Li[1*]

[1]Department of Laboratory Medicine, Renji Hospital, School of Medicine, Shanghai Jiaotong University, Shanghai, 200127, China.

[2]Division of Oncology, School of Medicine, Washington University in St. Louis, MO, 63110, USA.

[*]Corresponding author:

Min Li, Department of Laboratory Medicine, Renji Hospital, School of Medicine, Shanghai Jiaotong University

160 Pujian Road, Shanghai 200127, China

Phone: 86-21-68383614; Fax: 86-21-68383617

E-mail: minli__0075@126.com

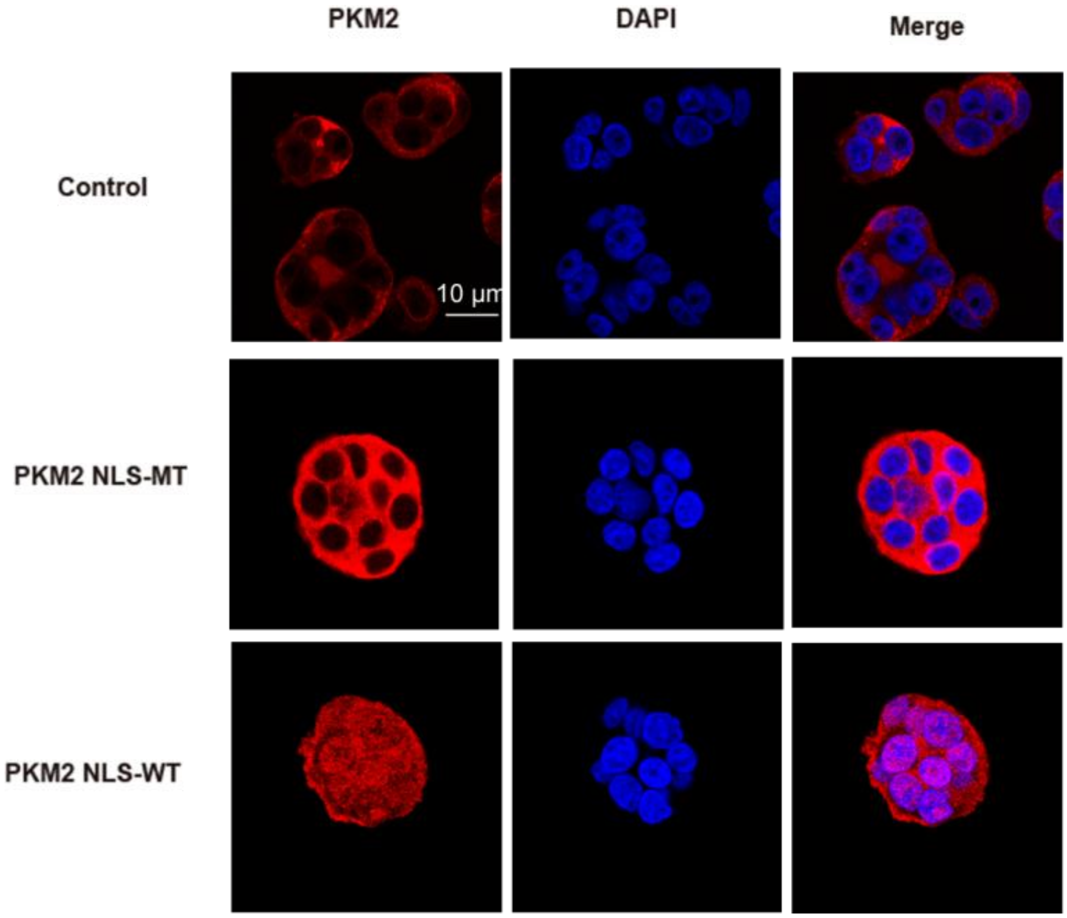

Supplementary Figure S1. Subcellular localization of PKM2-NLS, and PKM2-NLS mutant proteins in HT29 cells were detected by immunofluorescence (original magnification, 400x).

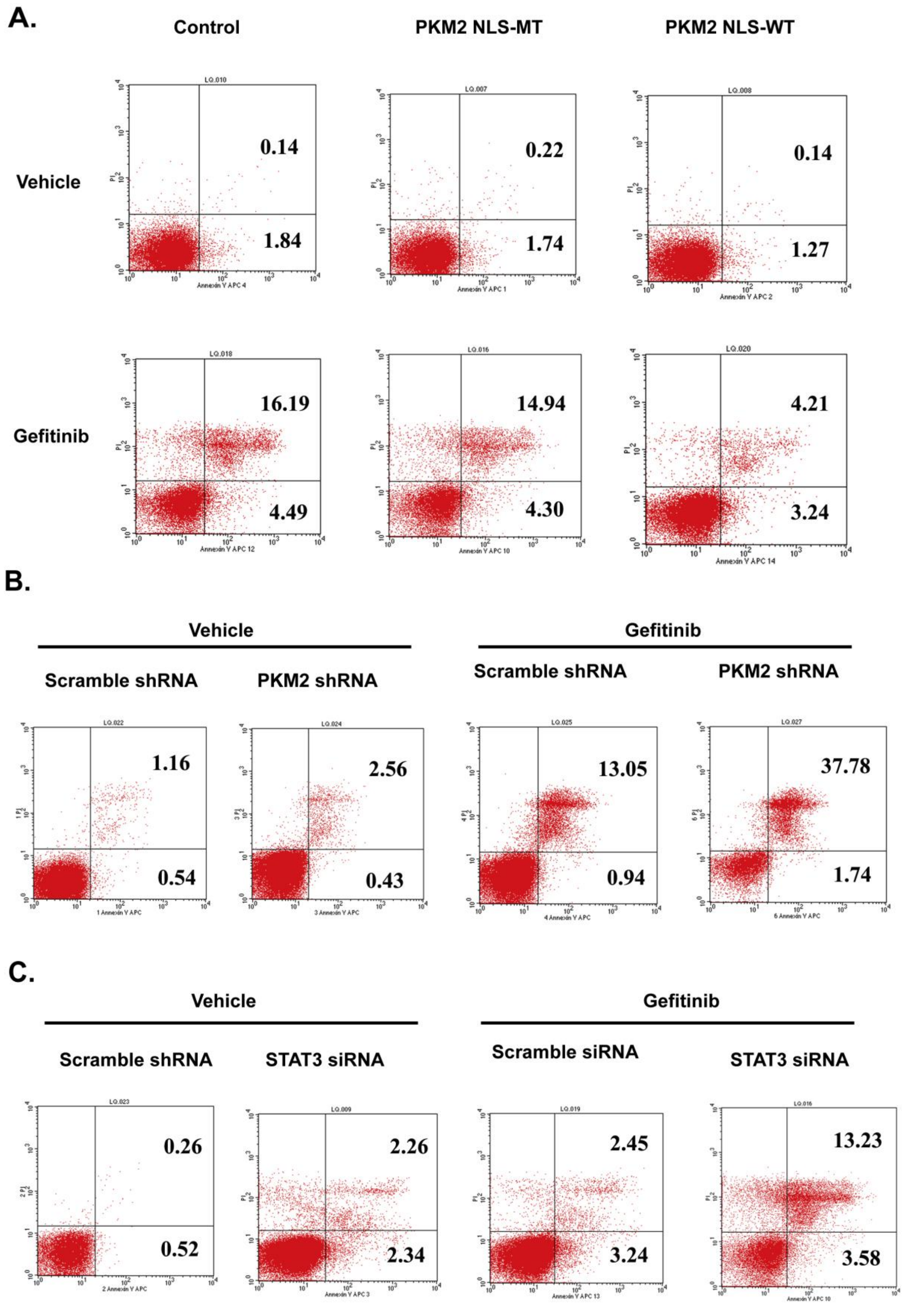

Supplementary Figure S2. Representative analysis of gefitinib-induced apoptosis.

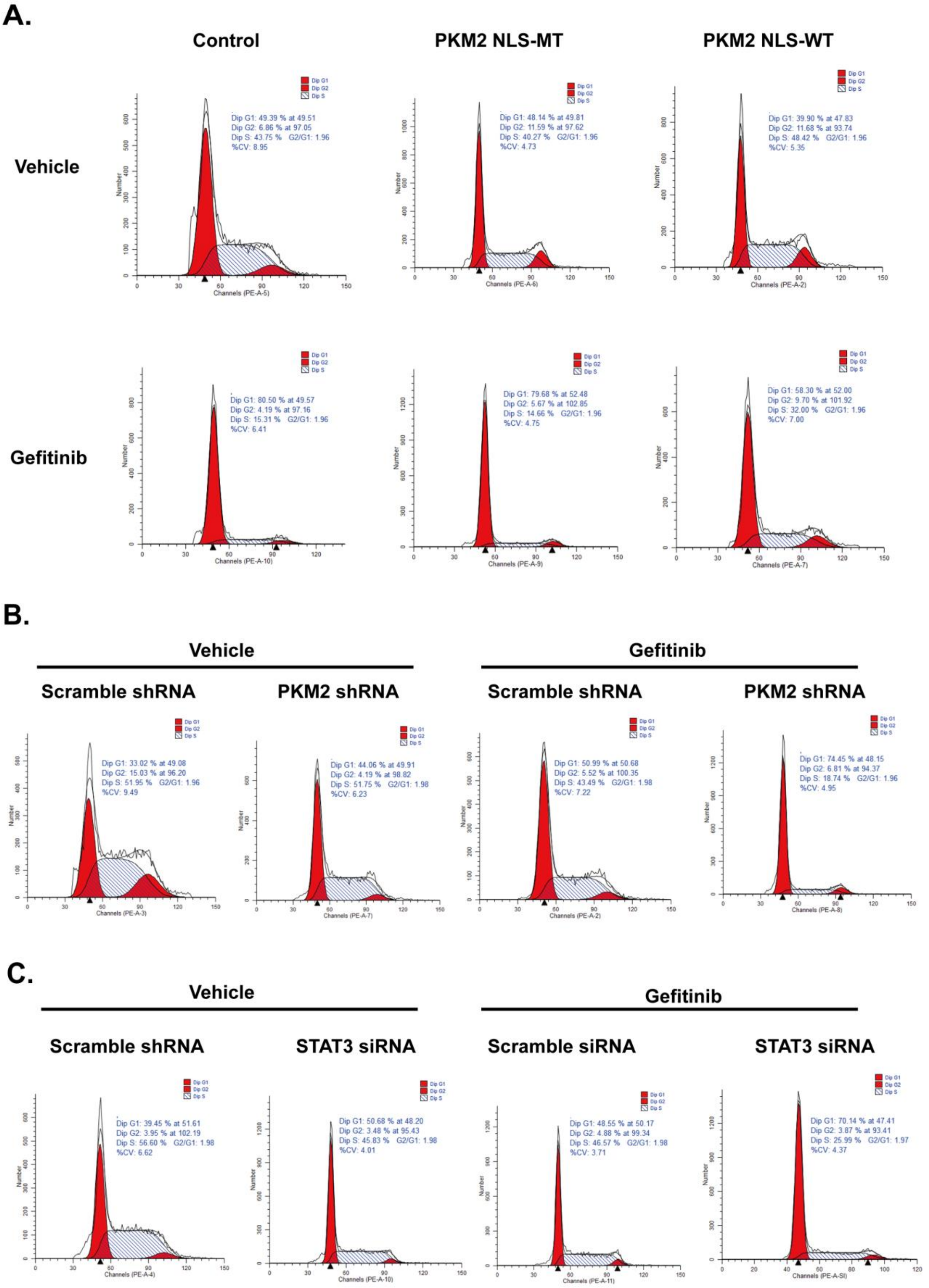

Supplementary Figure S3. Representative analysis of gefitinib-induced cell cycle arrest.

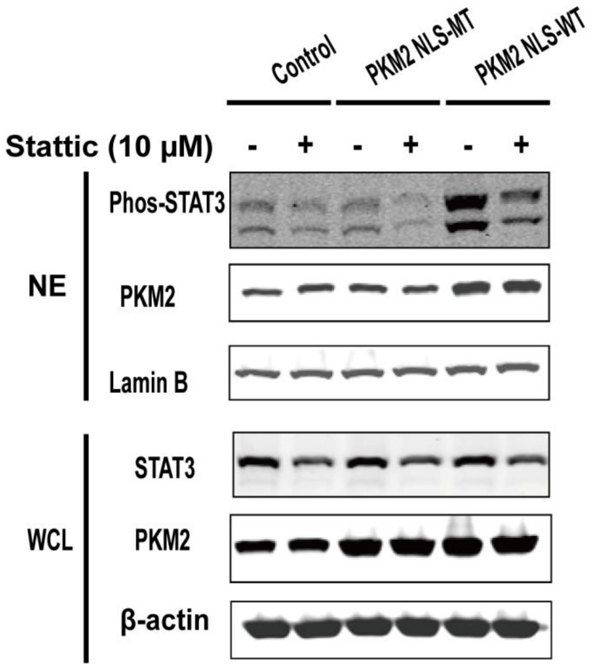

Supplementary Figure S4. Stattic inhibited STAT3 phosphorylation and expression in colon cancer cells

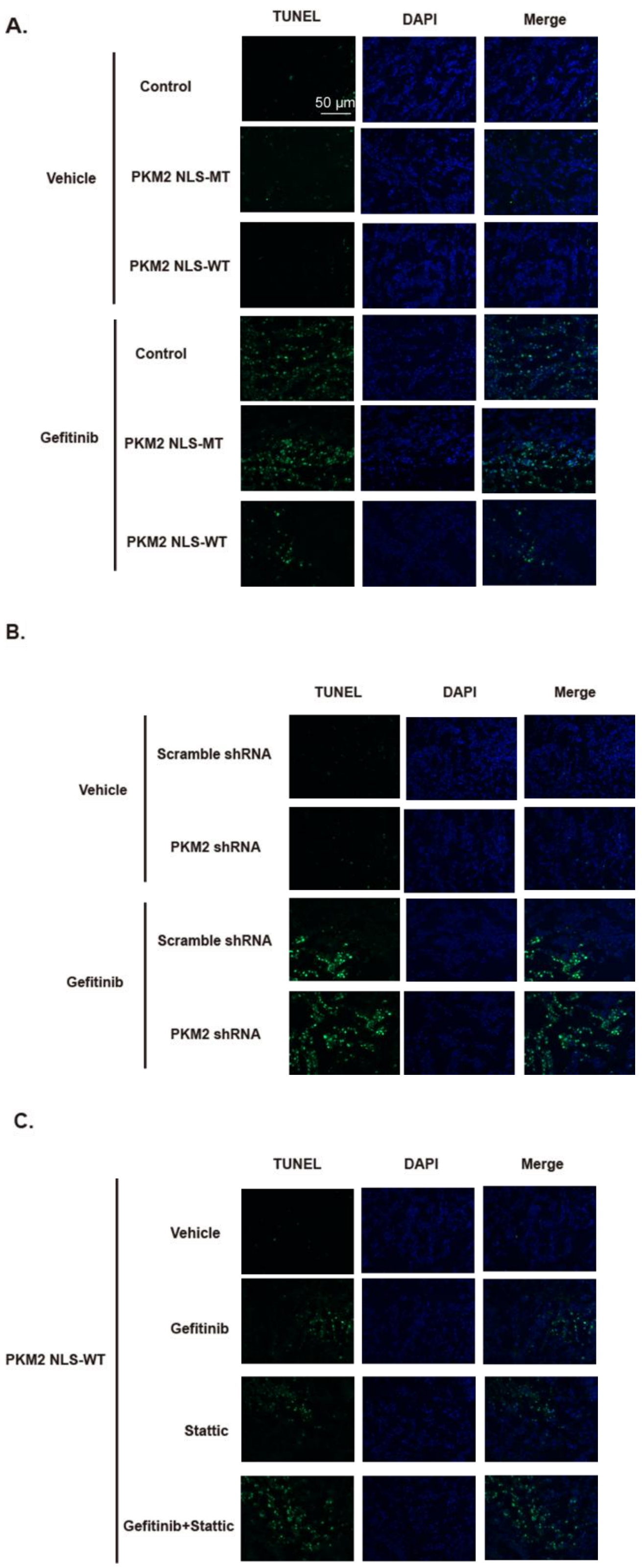

Supplementary Figure S5. Representative figures of apoptotic index induced by gefitinib in xenograft tumors (original magnification, 400x).

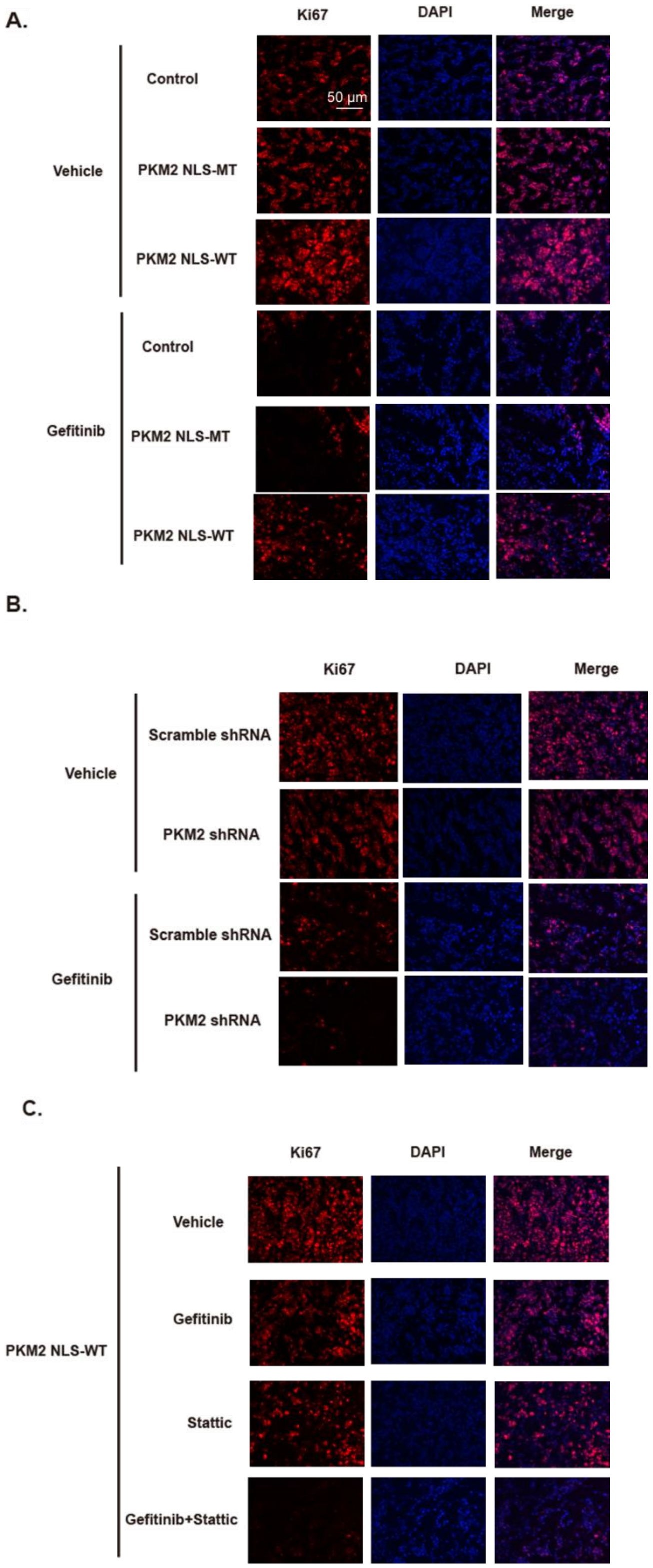

Supplementary Figure S6. Representative figures of proliferating index inhibited by gefitinib in xenograft tumors (original magnification, 400x).
